# Investigating the complementary value of OCT to MRI in cognitive impairment in relapsing-remitting multiple sclerosis

**DOI:** 10.1177/13524585241304356

**Published:** 2024-12-20

**Authors:** Ronja Christensen, Amy Jolly, Charmaine Yam, Marios C Yiannakas, Ahmed T Toosy, Marco Pitteri, Anna He, Riccardo Nistri, Suraya Mohamud, Eirini Samdanidou, Alan J Thompson, Olga Ciccarelli

**Affiliations:** Queen Square MS Centre, Department of Neuroinflammation, UCL Queen Square Institute of Neurology, Faculty of Brain Sciences, University College London, London, UK; Queen Square MS Centre, Department of Neuroinflammation, UCL Queen Square Institute of Neurology, Faculty of Brain Sciences, University College London, London, UK; Queen Square MS Centre, Department of Neuroinflammation, UCL Queen Square Institute of Neurology, Faculty of Brain Sciences, University College London, London, UK; Neurosciences Institute, Cleveland Clinic London, London, UK; National Institute for Health and Care Research, University College London Hospitals Biomedical Research Centre, London, UK; Queen Square MS Centre, Department of Neuroinflammation, UCL Queen Square Institute of Neurology, Faculty of Brain Sciences, University College London, London, UK; National Institute for Health and Care Research, University College London Hospitals Biomedical Research Centre, London, UK; Queen Square MS Centre, Department of Neuroinflammation, UCL Queen Square Institute of Neurology, Faculty of Brain Sciences, University College London, London, UK; National Institute for Health and Care Research, University College London Hospitals Biomedical Research Centre, London, UK; Queen Square MS Centre, Department of Neuroinflammation, UCL Queen Square Institute of Neurology, Faculty of Brain Sciences, University College London, London, UK; Department of Neuropsychology, National Hospital for Neurology and Neurosurgery, London, UK; Queen Square MS Centre, Department of Neuroinflammation, UCL Queen Square Institute of Neurology, Faculty of Brain Sciences, University College London, London, UK; National Institute for Health and Care Research, University College London Hospitals Biomedical Research Centre, London, UK; Clinical Neuroscience, Karolinska Institute, Stockholm, Sweden; Queen Square MS Centre, Department of Neuroinflammation, UCL Queen Square Institute of Neurology, Faculty of Brain Sciences, University College London, London, UK; Queen Square MS Centre, Department of Neuroinflammation, UCL Queen Square Institute of Neurology, Faculty of Brain Sciences, University College London, London, UK; Queen Square MS Centre, Department of Neuroinflammation, UCL Queen Square Institute of Neurology, Faculty of Brain Sciences, University College London, London, UK; Queen Square MS Centre, Department of Neuroinflammation, UCL Queen Square Institute of Neurology, Faculty of Brain Sciences, University College London, London, UK; National Institute for Health and Care Research, University College London Hospitals Biomedical Research Centre, London, UK; Queen Square MS Centre, Department of Neuroinflammation, UCL Queen Square Institute of Neurology, Faculty of Brain Sciences, University College London, London, UK; National Institute for Health and Care Research, University College London Hospitals Biomedical Research Centre, London, UK

**Keywords:** Cognition, optical coherence tomography (OCT), magnetic resonance imaging (MRI), Brief International Cognitive Assessment for MS (BICAMS)

## Abstract

**Background:**

Cognitive decline in multiple sclerosis (MS) is associated with neuro-axonal loss, quantifiable by optical coherence tomography (OCT). Associations between OCT measures and cognition in relapsing-remitting MS (RRMS) remain incompletely investigated, particularly the added value of OCT when combined with magnetic resonance imaging (MRI). We investigated the contributions of OCT and MRI while applying stringent criteria to control for subclinical optic neuropathy.

**Methods:**

In this cross-sectional study, 137 RRMS patients underwent OCT, Brief International Cognitive Assessment for MS (BICAMS), Expanded Disability Status Scale (EDSS) and brain MRI (lesion load, grey and white matter volume); associations were explored using linear regression models.

**Results:**

RRMS patients (aged 40.88 ± 10.6 years; disease duration 7.95 ± 7.39 years; EDSS 2; 0–6.5) were studied. Of BICAMS, 50.36% showed impaired Symbol Digit Modalities Test (SDMT), 37.23% impaired Brief Visuospatial Memory Test and 5.11% impaired California Verbal Learning and Memory Test; better SDMT performance was associated with thicker ganglion cell-inner plexiform (GCIPL) layers for eyes unaffected by optic neuritis (*B* = 0.23, 95% CI = (0.01–0.44), *p* = 0.03), but not when MRI measures were included (*B* = 0.18, CI = (−0.03 to 0.38), *p* = 0.09).

**Conclusion:**

GCIPL thinning correlates with SDMT, supporting OCT as a biomarker of cognitive dysfunction. However, GCIPL did not uniquely predict SDMT performance when including MRI measures, suggesting limited utility of OCT in predicting cognitive performance over MRI in RRMS.

## Introduction

Multiple sclerosis (MS) is a chronic neurological disorder characterised by demyelination and neurodegeneration resulting in physical and cognitive disability.^1^ Cognitive impairment can be present at all stages and subtypes of the disease, affecting 40%–70% of patients^2^ and is linked to higher unemployment rates, and lower quality of life.^3^

Magnetic resonance imaging (MRI) studies have demonstrated associations between cognitive decline and brain atrophy in MS patients, underscoring neurodegeneration as a key driver of cognitive decline, in addition to inflammation and demyelination.^4^ Recently, optical coherence tomography (OCT) has gained attention as a potential tool for monitoring neurodegeneration in MS. OCT estimates the thickness of the retinal nerve fibre layer (RNFL), which is composed of the unmyelinated axons of retinal ganglion cells, and the ganglion cell-inner plexiform layer (GCIPL), which contains the cell bodies of the retinal ganglion cells and their synapses. Specifically, in the absence of damage, such as optic neuritis (ON), the RNFL and GCIPL are thought to reflect central nervous system (CNS) structure.^5^

Inner retinal thinning has been linked to brain atrophy, notably grey matter (GM), but also white matter (WM) and lesion volumes.^6^ Moreover, several studies have reported associations between thinning on retinal OCT measures and worse cognitive performance,^7–11^ supporting the role of OCT-derived measures as indirect biomarkers of CNS degeneration. However, other studies did not confirm these findings.^12,13^ These inconsistent results relate in part to the inclusion of mixed clinical subtypes, and different cognitive domains assessed across studies. Importantly, given that the role of OCT as a mirror of CNS degeneration depends strictly on the absence of ON-related thinning, differences in classifications of affected and non-affected eyes may further contribute to previous inconsistent findings. Indeed, Davion et al.^14^ employed MRI with double inversion recovery acquisition to account for subclinical ON and found that the association between OCT and clinical parameters was just a reflection of asymptomatic ON, thus mandating a stricter approach to the classification of non-affected eyes when evaluating OCT as a measure of CNS neurodegeneration.

It also remains unclear whether OCT measures offer unique explanatory variance complementary to MRI. In clinical practice, where time and resources are limited, it might be prudent to determine the utility of adding OCT to MRI to explain cognitive dysfunction.

This study aimed to (1) establish the association between OCT measures and cognitive performance in relapsing-remitting MS (RRMS) patients and (2) investigate the added value of OCT when used with MRI in explaining cognitive performance. We employed strict methods to classify non-affected eyes by taking recent guidelines on inter-eye difference (IED) cut-off points into consideration.^15^ We used the Brief International Cognitive Assessment for MS (BICAMS) to assess cognitive performance.^16^

## Methods

### Patients

We recruited consecutive RRMS patients who initiated a disease-modifying therapy (DMT) at the National Hospital for Neurology and Neurosurgery (NHNN), University College London, United Kingdom.

Inclusion criteria were as follows: (1) clinically definite RRMS diagnosis according to the 2017 McDonald Criteria^17^ and (2) within 3 months from initiation of a new DMT for MS at the NHNN. Exclusion criteria were as follows: (1) clinical history of bilateral ON; (2) known ophthalmological comorbidities such as glaucoma,^18^ as per the OSCAR-IB criteria; (3) refractive errors >6 or <−6 dioptres; (4) use of steroid therapy in the last 3 months; (5) history of learning disability or major psychiatric conditions; and (6) use of cognitively altering drugs, such as antidepressants or lisdexamfetamine.

Ethical approval for the study was granted by the Health Research Authority (HRA), and Health and Care Research Wales (HCRW) on 21 June 2019 (19/WA/0157; IRAS code: 257366, EDGE number: 121353). All patients provided written informed consent.

### Procedure

Patients were invited for a study visit at baseline. Clinical data were collected through patient interviews and review of medical records including demographics, disease duration, DMT, relapse history and ON history. All participants underwent cognitive testing, physical examination, MRI and OCT (see subsequent sections). We aimed to complete all assessments on the same day, but where this was not possible, a 3-month delay was accepted.

### Cognitive and clinical assessments

Patients underwent cognitive testing with the BICAMS,^16^ which comprised: (1) Symbol Digit Modalities Test (SDMT), measuring processing speed; (2) the first three recall trials of the Brief Visuospatial Memory Test Revised (BVMT-R), measuring visual learning memory; and (3) the first five trials of the California Verbal Learning Test, Second Edition (CVLT-II), measuring verbal learning.

Age, sex and years of education were used to convert raw in *z*-scores for each participant according to available normative data.^19^ A test score was considered impaired if below 1.5 standard deviations from the mean. Patients’ physical disability was assessed by a trained neurologist using the Expanded Disability Status Scale (EDSS).^20^

### MRI protocol

MRI was performed using a 3 Tesla Philips Ingenia CX MRI system (Philips Medical Systems, Best, Netherlands) with the standard NV 16-channel coil. The brain MRI protocol is reported in Supplemental Material (S1).

Images were pre-processed as follows: lesions were defined using the automated lesion delineation software, nicMS,^21^ using three-dimensional (3D) T2-FLAIR images as input. All lesion masks were manually inspected and corrected where necessary, and lesion load was calculated as the total volume of focal lesions. Lesion filling of 3D T1-Turbo Field Echo (TFE) images was then performed, and subsequently, images were segmented into GM and WM tissue using the Geodesic Information Flow software^22^ and tissue volumes (mm^3^) were extracted using NiftySeg software. All tissue volumes were corrected by total intracranial volume.

### OCT

The OCT protocol is reported following the Advised Protocol for OCT Study Terminology and Elements recommendations (APOSTEL) version 2.0.^23^

Retinal images were acquired in a dark room by trained operators using a Spectral Domain (SD)-OCT machine (Heidelberg Spectralis, software V6.16.2). The OCT scanning protocol is reported in Supplemental Material (S2).^18^

**Figure 1.**
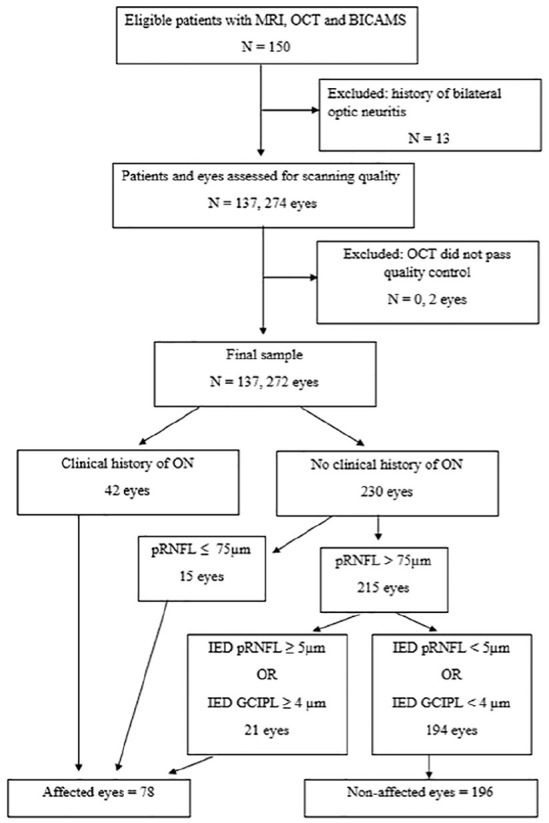
Flowchart of patient selection and categorisation of eyes. MRI: Magnetic resonance imaging; OCT: optical coherence tomography; BICAMS: Brief International Cognitive Assessment for MS; ON: optic neuritis; pRNFL: peripapillary retinal nerve fibre layer; GCIPL: ganglion cell-inner plexiform layer; IED: inter-eye differences. Where IED were larger than 5 μm for pRNFL or 4 μm for GCIPL, the thicker eye was included in the non-affected category and provided that the pRNFL was larger than 75 μm.

Information about ON history was obtained by patient interviews and review of medical records. Patients with bilateral ON history were excluded. We sought to prioritise identification of unaffected eyes, and therefore, we applied an absolute threshold of peripapillary retinal nerve fibre layer (pRNFL) and an IED threshold. We used published normative data^24^ to threshold pRNFL values of ⩽75 µm to signify optic neuropathy at a 99% confidence interval. For patients with a clinical history of unilateral ON, the other eye was considered unaffected where pRNFL > 75 µm. For eyes without ON history, we applied stringent criteria to ensure that unaffected eyes were not contaminated with affected eyes: pRNFL > 75 µm, IED of pRNFL ⩽ 5 µm or GCIPL ⩽ 4 µm.^15^ For eyes with a clinical history of ON, 28 out of 43 (65.11%) showed pRNFL > 75 µm, while 95% violated the IED criteria. For patients contributing both eyes to the same category, we averaged the values of both eyes to ensure that all patients contributed only one value to the analyses. In total, 67 patients had both eyes unaffected, while 8 had both eyes affected (Figure 1).

### Statistical analysis

Statistical analyses were performed using Rstudio (version: 2023.06.0). Continuous variables were expressed as means and standard deviations, or median and range, and categorical variables as numbers and percentages. Data distribution was assessed using the Shapiro–Wilks test. Pearson’s and Spearman’s tests of univariate correlations were used for parametric and non-parametric variables. Multivariable linear regression models were used to assess the explanatory value (quantified by adjusted *R*^2^) of OCT measures and MRI measures (alone and combined) on cognitive and physical disability variables. Cognitive (BICAMS) and physical (EDSS) scores were treated as dependent variables in separate models, while OCT (pRNFL and GCIPL) and MRI (WM, GM and lesion volume) measures along with potential confounding variables (age, sex, disease duration and years of education) were considered independent variables. To ensure that multicollinearity was not influencing regression results, we computed the variance inflation factor (VIF) for each predictor in the models. A VIF value below 5 was used as a threshold to determine the presence of significant multicollinearity. All predictors in both the OCT and MRI models had VIF values well below this threshold, indicating low collinearity. A model comparison approach using analysis of variance (ANOVA) was used to compare models including only MRI measures as independent variables with models including both MRI and OCT measures. Only results associated with *p* < 0.05 were considered statistically significant and, therefore, reported in the results.

## Results

### Patient characteristics

This study included 150 patients with RRMS but 13 were excluded due to clinically confirmed bilateral ON. Demographic and clinical characteristics are reported in Table 1. The sample comprised mainly female (69.34%) middle-aged adults (40.88 ± 10.6 years), with relatively short median disease duration (7.95 ± 7.39 years) and low physical disability (median, EDSS = 2, range, 0–6.5). None of the patients had a diagnosis of vascular or other neurodegenerative conditions. In terms of cognitive performance, 50.36% of the patients scored below the cut-off (i.e., *z*-score deviation greater than 1.5) on the SDMT and 37.23% on the BVMT-R, while few patients (*N* = 7, 5.11%) scored below the cut-off on the CVLT-II.

**Table 1.** Participant characteristics.

	*N* = 137
Age, years, mean (*SD*)	40.88 (10.6)
Sex, females *N* (%)	95 (69.34)
Ethnicity, white *N* (%)	97 (70.8)
Years of education, mean (*SD*)	15.47 (2.51)
Disease duration years, mean (*SD*)	7.95 (7.39)
EDSS, median (range)	2 (0–6.5)
BICAMS, mean (*SD*) (% impairment)	
SDMT	52.67 (13.44) (50.36%)
BVMT-R	23.97 (7.48) (37.23%)
CVLT-II	52.27 (10.95) (5.11%)
Normalised MRI measures, mean (*SD*)	
White matter volume	0.30 (0.01)
Grey matter volume	0.44 (0.01)
Lesion volume	0.01 (0.01)
Retinal OCT measures, mean (*SD*) (µm)	
Affected eyes’ pRNFL	82.75 (14.78)
Affected eyes’ GCIPL	71.64 (14.13)
Unaffected eyes’ pRNFL	96.61 (9.71)
Unaffected eyes’ GCIPL	86.67 (10.59)

EDSS: Expanded Disability Status Scale; BICAMS: Brief International Cognitive Assessment for Multiple Sclerosis; SDMT: Symbol Digit Modalities Test; BVMT-R: Brief Visuospatial Memory Test Revised; CVLT-II: California Verbal Learning Test, Second Edition; MRI: magnetic resonance imaging; OCT: optical coherence tomography; pRNFL: peripapillary retinal nerve fibre layer; GCIPL: ganglion cell-inner plexiform layer.

Most patients (*n* = 120, 87.5%) completed all assessments on the same day, while 13 had up to a month interval between assessments (9.5%) and a few had assessments spread over 3 months (*n* = 4, 3%). No patients experienced relapses between assessments.

### Univariate correlations between OCT, MRI and BICAMS

In terms of MRI, greater WM and GM volume and lower lesion volume correlated with higher scores on the SDMT, BVMT-R, CVLT-II and lower EDSS, except for the CVLT-II, which showed no correlation with GM volume (*r* = 0.14, *p* = 0.10) (Table 2).

**Table 2.** Correlations between clinical and imaging variables.

	SDMT	BVMT-R	CVLT-II	EDSS	WM	GM	Lesion	RNFL affected	RNFL unaffected	GCIPL affected	GCIPL unaffected
SDMT	1										
BVMT-R	**0.65**, *p* **<** **0.001**	1									
CVLT-II	**0.54**, *p* **<** **0.001**	**0.64**, *p* **<** **0.001**	1								
EDSS	−**0.59**, *p* **<** **0.001**	−**0.54**, *p* **<** **0.001**	−**0.39**, *p* **<** **0.001**	1							
WM	**0.35**, *p* **<** **0.001**	**0.34**, *p* **<** **0.001**	**0.19**, *p* = **0.02**	−**0.28**, *p* **<** **0.001**	1						
GM	**0.30**, *p* **<** **0.001**	**0.30**, *p* **<** **0.001**	0.14, *p* = 0.10	−**0.25**, *p* **<** **0.01**	−0.11, *p* = 0.18	1					
Lesion	−**0.43**, *p* **<** **0.001**	−**0.39**, *p* **<** **0.001**	−**0.27**, *p* **<** **0.001**	**0.22**, *p* **<** **0.01**	−**0.39**, *p* **<** **0.001**	−**0.36**, *p* **<** **0.001**	1				
RNFL all eyes	**0.18**, *p* = **0.04**	0.15, *p* = 0.08	0.05, *p* = 0.6	−**0.2**, *p* = **0.02**	**0.3**, *p* **<** **0.001**	**0.18**, *p* = **0.04**	−0.13, *p* = 0.14				
RNFL affected	0.09, *p* = 0.49	0.02, *p* = 0.84	−0.04, *p* = 0.75	−0.15, *p* = 0.21	0.11, *p* = 0.34	0.07, *p* = 0.54	0.01, *p* = 0.91	1			
RNFL unaffected	0.13, *p* = 0.16	**0.17**, *p* = **0.05**	0.08, *p* = 0.38	−**0.24**, *p* **<** **0.01**	**0.24**, *p* **<** **0.01**	0.08, *p* = 0.39	−0.07, *p* = 0.39	**0.76**, *p* **<** **0.001**^a^	1		
GCIPL all eyes	**0.26**, *p* **<** **0.01**	0.17, *p* = 0.05	0.01, *p* = 0.9	−**0.19**, *p* = **0.03**	**0.3**, *p* **<** **0.001**	**0.22**, *p* = **0.01**	−**0.32**, *p* **<** **0.001**				
GCIPL affected	0.19, *p* = 0.13	0.03, *p* = 0.83	−0.01, *p* = 0.91	−0.18, *p* = 0.13	0.19, *p* = 0.13	−0.01, *p* = 0.93	−0.05, *p* = 0.68	**0.75**, *p* **<** **0.001**	**0.48**, *p* **<** **0.001**^a^	1	
GCIPL unaffected	**0.27**, *p* **<** **0.01**	0.12, *p* = 0.19	0.03, *p* = 0.76	−0.13, *p* = 0.15	**0.22**, *p* = **0.01**	0.09, *p* = 0.29	−**0.19**, *p* = **0.04**	**0.45**, *p* **<** **0.001**^a^	**0.53**, *p* **<** **0.001**	**0.68**, *p* **<** **0.001**^a^	1

SDMT: Symbol Digit Modalities Test; BVMT-R: Brief Visuospatial Memory Test Revised; CVLT-II: California Verbal Learning Test, Second Edition; EDSS: Expanded Disability Status Scale; WM: white matter; GM: grey matter; RNFL: retinal nerve fibre layer; GCIPL: ganglion cell-inner plexiform layer. Bold font indicates statistical significance at the p < 0.05 level.

a Correlation analyses included only patients who contributed an eye to each group (*N* = 61).

**Figure 2.**
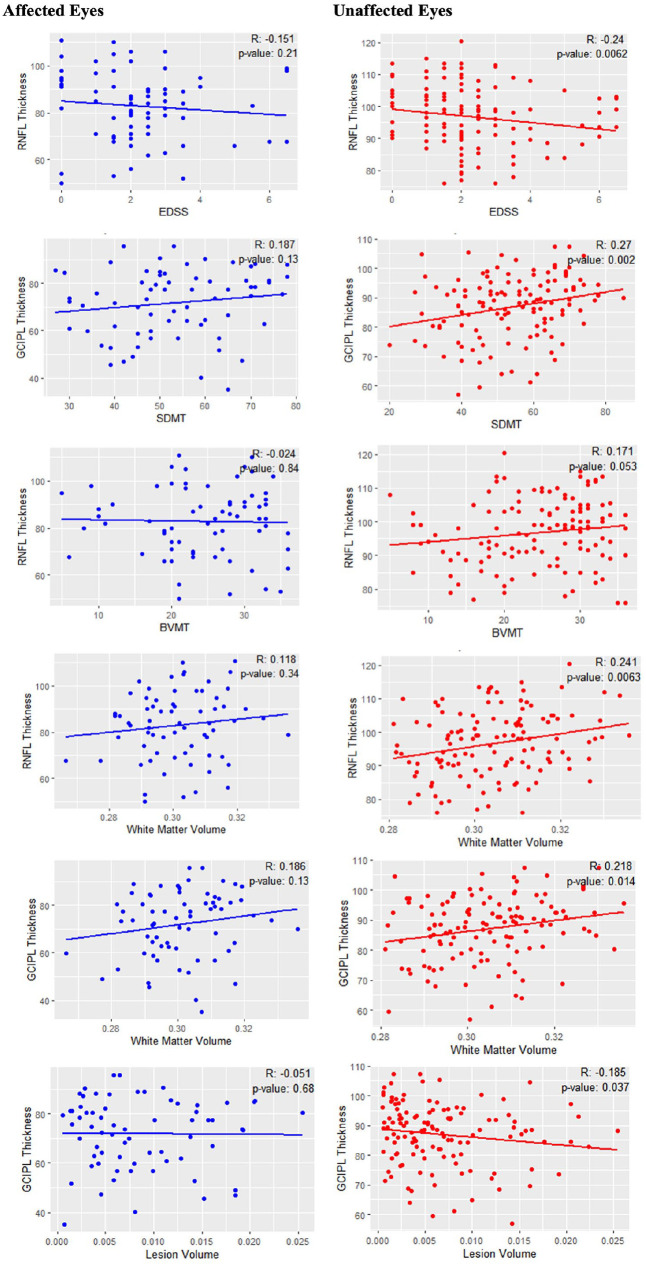
Scatterplots of correlations between imaging and clinical variables for affected (blue) and non-affected (red) eyes. RNFL: retinal nerve fibre layer; EDSS: Expanded Disability Status Scale; GCIPL: ganglion cell-inner plexiform layer; SDMT: Symbol Digit Modalities Test; BVMT: Brief Visuospatial Memory Test.

When dividing eyes by ON history, GCIPL thickness of non-affected eyes correlated with better performance on the SDMT (*r* = 0.27, *p* < 0.01), higher WM volume (*r* = 0.22, *p* < 0.01) and lower lesion volume (*r* = −0.19, *p* = 0.04); RNFL thickness of non-affected eyes correlated with better performance on the BVMT-R (*r* = 0.19, *p* = 0.05), lower EDSS (*r* = −0.24, *p* < 0.01) and higher WM volume (*r* = 0.24, *p* < 0.01) (Figure 2). Correlations for affected eyes and for all eyes irrespective of categorisation are reported in Figure 2.

### Multivariable analyses

Multivariable analyses adjusted for age, sex, disease duration and years of education (for the BICAMS) were performed. A test for multicollinearity using the VIF showed no evidence of significant multicollinearity (all VIF < 1.8). These analyses yielded non-significant results for OCT measures in affected eyes on all BICAMS subtests and EDSS (all *p* > 0.16) (Table 3).

**Table 3.** Multivariate analyses of OCT metrics and cognitive performance and EDSS.

	SDMT	BVMT-R	CVLT-II	EDSS
Models with affected RNFL				
RNFL thickness	*B* = −0.06 CI = [−0.29 to 0.18] *p* = 0.65	*B* = −0.09 CI = [−0.22 to 0.05] *p* = 0.21	*B* = −0.13 CI = [−0.33 to 0.06] *p* = 0.17	*B* = 0.00 CI = [−0.03 to 0.03] *p* = 0.97
Age	*B* = −0.19 CI = [−0.52 to 0.14] *p* = 0.25	*B* = −0.10 CI = [−0.28 to 0.08] *p* = 0.28	*B* = −0.11 CI = [−0.38 to 0.15] *p* = 0.39	*B* = 0.02 CI = [−0.02 to 0.06] *p* = 0.34
Sex	*B* = −1.94 CI = [−9.24 to 5.35] *p* = 0.59	*B* = −2.63 CI = [−6.73 to 1.46] *p* = 0.20	*B* = −5.54 CI = [−11.43 to 0.36] *p* = 0.07	*B* = **0.90** CI = [0.05 to 1.75] *p* = 0.04
Disease duration	*B* = −0.03 CI = [−0.07 to 0.01] *p* = 0.09	*B* = −**0.02** CI = [−0.04 to −0.00] *p* = 0.04	*B* = −0.01 CI = [−0.05 to 0.02] *p* = 0.37	*B* = 0.00 CI = [−0.00 to 0.01] *p* = 0.75
Years of education	*B* = 0.57 CI = [−0.87 to 2.00] *p* = 0.43	*B* = −0.06 CI = [−0.86 to 0.75] *p* = 0.88	*B* = 0.06 CI = [−1.10 to 1.22] *p* = 0.91	–
Model estimates	*F*(5,63) = 1.37, *p* = 0.25, adj. *R*^2^ = 0.03	*F*(5,63) = 2.12, *p* = 0.07, adj. *R*^2^ = 0.08	*F*(5,63) = 1.26, *p* = 0.29, adj. *R*^2^ = 0.02	*F*(4,64) = 1.62, *p* = 0.18, adj. *R*^2^ = 0.04
Models with unaffected RNFL				
RNFL thickness	*B* = 0.01 CI = [−0.23 to 0.25] *p* = 0.94	*B* = 0.02 CI = [−0.11 to 0.16] *p* = 0.74	*B* = −0.09 CI = [−0.28 to 0.10] *p* = 0.33	*B* = −0.02 CI = [−0.05 to 0.01] *p* = 0.29
Age	*B* = −**0.29** CI = [−0.52 to −0.06] *p* = 0.02	*B* = −**0.15** CI = [−0.28 to −0.02] *p* = 0.02	*B* = −0.11 CI = [−0.29 to 0.07] *p* = 0.24	*B* = **0.05** CI = [0.03 to 0.08] *p* < 0.001
Sex	*B* = −2.60 CI = [−7.58 to 2.38] *p* = 0.30	*B* = −**3.28** CI = [−6.09 to −0.46] *p* = 0.02	*B* = −**7.77** CI = [−11.72 to −3.81] *p* < 0.001	*B* = 0.53 CI = [−0.10 to 1.16] *p* = 0.10
Disease duration	*B* = −**0.03** CI = [−0.05 to 0.00] *p* = 0.05	*B* = −0.01 CI = [−0.03 to 0.00] *p* = 0.12	*B* =−0.01 CI = [−0.04 to 0.01] *p* = 0.22	*B* = −0.00 CI = [−0.00 to 0.00] *p* = 0.39
Years of education	*B* = **1.20** CI = [0.31 to 2.09] *p* = 0.01	*B* = 0.29 CI = [−0.21 to 0.80] *p* = 0.25	*B* = **1.06** CI = [0.35 to 1.76] *p* = 0.004	–
Model estimates	*F* **(5,123)** = **4.56**, *p* **<** **0.001, adj. *R***^2^ = **0.12**	*F* **(5,123)** = **3.83**, *p* = **0.003, adj. *R***^2^ = **0.10**	*F* **(5,123)** = **5.26**, *p* **<** **0.001, adj. *R***^2^ = **0.14**	*F* **(4,124)** = **5.42**, *p* **<** **0.001, adj. *R***^2^ = **0.12**
Models with affected GCIPL				
GCIPL thickness	*B* = 0.04 CI = [−0.20 to 0.29] *p* = 0.73	*B* = −0.10 CI = [−0.24 to 0.04] *p* = 0.16	*B* = −0.11 CI = [−0.31 to 0.09] *p* = 0.27	*B* = 0.00 CI = [−0.03 to 0.03] *p* = 0.97
Age	*B* = −0.14 CI = [−0.47 to 0.19] *p* = 0.39	*B* = −0.10 CI = [−0.29 to 0.08] *p* = 0.27	*B* = −0.09 CI = [−0.36 to 0.17] *p* = 0.48	*B* = **0.02** CI = [−0.02 to 0.06] *p* = 0.36
Sex	*B* = −1.98 CI = [−9.30 to 5.33] *p* = 0.59	*B* = −2.96 CI = [−7.02 to 1.11] *p* = 0.15	*B* = −**5.95** CI = [−11.81 to −0.09] *p* = 0.05	*B* = **0.95** CI = [0.09 to 1.80] *p* = 0.03
Disease duration	*B* = −0.03 CI = [−0.07 to 0.01] *p* = 0.09	*B* = −**0.02** CI = [−0.05 to −0.00] *p* = 0.03	*B* = −0.02 CI = [−0.05 to 0.02] *p* = 0.33	*B* = 0.00 CI = [−0.00 to 0.01] *p* = 0.69
Years of education	*B* = 0.44 CI = [−0.98 to 1.87] *p* = 0.54	*B* = −0.17 CI = [−0.97 to 0.62] *p* = 0.66	*B* = −0.13 CI = [−1.28 to 1.01] *p* = 0.82	–
Model estimates	*F*(5,62) = 1.44, *p* = 0.22, adj. *R*^2^ = 0.03	*F* **(5,62)** = **2.46**, *p* = **0.04, adj. *R***^2^ = **0.09**	*F*(5,62) = 1.4, *p* = 0.24, adj. *R*^2^ = 0.03	*F*(4,63) = 1.69, *p* = 0.16, adj. *R*^2^ = 0.04
Models with unaffected GCIPL				
GCIPL thickness	*B* = **0.23** CI = [0.02 to 0.44] *p* = 0.03	*B* = 0.05 CI = [−0.07 to 0.17] *p* = 0.43	*B* = −0.02 CI = [−0.19 to 0.15] *p* = 0.83	*B* = −0.00 CI = [−0.03 to 0.02] *p* = 0.73
Age	*B* = −**0.23** CI = [−0.46 to −0.01] *p* = 0.05	*B* = −**0.15** CI = [−0.28 to −0.01] *p* = 0.03	*B* = −0.10 CI = [−0.28 to 0.09] *p* = 0.31	*B* = **0.06** CI = [0.03 to 0.09] *p* < 0.001
Sex	*B* = −3.01 CI = [−7.83 to 1.81] *p* = 0.22	*B* = −**3.44** CI = [−6.21 to −0.67] *p* = 0.02	*B* = −**7.38** CI = [−11.30 to −3.47] *p* < 0.001	*B* = 0.60 CI = [−0.03 to 1.22] *p* = 0.06
Disease duration	*B* = −0.03 CI = [−0.05 to 0.00] *p* = 0.06	*B* = −0.01 CI = [−0.03 to 0.00] *p* = 0.12	*B* = −0.01 CI = [−0.03 to 0.01] *p* = 0.26	*B* = −0.00 CI = [−0.00 to 0.00] *p* = 0.45
Years of education	*B* = **1.18** CI = [0.31 to 2.05] *p* = 0.01	*B* = 0.30 CI = [−0.20 to 0.80] *p* = 0.24	*B* = **1.02** CI = [0.32 to 1.73] *p* = 0.01	–
Model estimates	*F* **(5,123)** = **5.67**, *p* **<** **0.001, adj. *R***^2^ = **0.15**	*F* **(5,123)** = **3.94**, *p* = **0.002, adj. *R***^2^ = **0.10**	*F* **(5,123)** = **5.04**, *p* **<** **0.001, adj. *R***^2^ = **0.14**	*F* **(4,124)** = **5.13**, *p* **<** **0.001, adj. *R***^2^ = **0.11**

SDMT: Symbol Digit Modalities Test; BVMT-R: Brief Visuospatial Memory Test Revised; CVLT-II: California Verbal Learning Test, Second Edition; EDSS: Expanded Disability Status Scale; RNFL: retinal nerve fibre layer; GCIPL: ganglion cell-inner plexiform layer. Bold font indicates statistical significance at the p < 0.05 level.

The association between GCIPL thickness of the non-affected eye and the SDMT remained significant (*B* = 0.23, 95% CI = (0.02–0.44), *p* = 0.03) (Table 3). On the contrary, RNFL thickness of the non-affected eye no longer remained a significant predictor of BVMT-R (*B* = 0.02, 95% CI = (−0.11 to 0.16), *p* = 0.74), but age and sex explained significant variance (*p* < 0.02).

In the multivariable analysis combining GCIPL with MRI measures (lesion, GM and WM volumes), the VIF values remained low (<1.8), suggesting no significant multicollinearity between predictors. This analysis revealed a significant combined explanatory value of the SDMT, *F*(8,118) = 6.80, *p* < 0.001, with an adjusted *R*^2^ of 0.27. However, GCIPL was not a significant predictor within the model (*B* = 0.18, 95% CI = (−0.03 to 0.38), *p* = 0.09). Moreover, when comparing the model including only MRI measures as predictors, *F*(7,119) = 7.25, *p* < 0.001, adjusted *R*^2^ = 0.26, with the model including both MRI measures and GCIPL, an ANOVA showed no significant differences, *F*(1,118) = 2.85, *p* = 0.10 (Table 4).

**Table 4.** Multivariable models of OCT and MRI measures with SDMT.

	Model 1: OCT	Model 2: MRI	Model 3: OCT + MRI
GCIPL thickness	*B* = **0.23** CI = [0.02–0.44] *p* = 0.03	–	*B* = 0.18 CI = [−0.03 to 0.38] *p* = 0.09
White matter volume	–	*B* = **308.73** CI = [105.08 to 512.39] *p* = 0.003	*B* = **286.33** CI = [82.52 to 490.14] *p* = 0.006
Grey matter volume	–	*B* = 265.68 CI = [−19.07 to 550.43] *p* = 0.07	*B* = 271.25 CI = [−11.41 to 553.91] *p* = 0.06
Lesion volume	–	*B* = −401.27 CI = [−883.75 to 81.21] *p* = 0.10	*B* = −384.01 CI = [−863.25 to 95.24] *p* = 0.12
Age	*B* = −**0.23** CI = [−0.46 to −0.01] *p* = 0.05	*B* = −0.20 CI = [−0.43 to 0.03] *p* = 0.08	*B* = −0.16 CI = [−0.39 to 0.07] *p* = 0.17
Sex	*B* = −3.01 CI = [−7.83 to 1.81] *p* = 0.22	*B* = −2.89 CI = [−7.54 to 1.76] *p* = 0.22	*B* = −3.02 CI = [−7.63 to 1.60] *p* = 0.19
Disease duration	*B* = −0.03 CI = [−0.05 to 0.00] *p* = 0.06	*B* = −0.01 CI = [−0.03 to 0.02] *p* = 0.58	*B* = −0.01 CI = [−0.03 to 0.02] *p* = 0.62
Years of education	*B* = **1.18** CI = [0.31 to 2.05] *p* = 0.008	*B* = **1.27** CI = [0.43 to 2.12] *p* = 0.003	*B* = **1.26** CI = [0.43 to 2.10] *p* = 0.003
Model estimates	*F* **(5,123)** = **5.67**, *p* **<** **0.001, adj. *R***^2^ = **0.15**	*F* **(7,119)** = **7.25**, *p* **<** **0.001, adj. *R***^2^ = **0.26**	*F* **(8,118)** = **6.80**, *p* **<** **0.001, adj. *R***^2^ = **0.27**

GCIPL: ganglion cell-inner plexiform layer.

Model 1 includes GCIPL; Model 2 includes MRI measures; and Model 3 includes both GCIPL and MRI measures. Bold font indicates statistical significance at the p < 0.05 level.

### Sensitivity analyses

We conducted sensitivity analyses in mildly (EDSS < 3, *N* = 90) versus severely disabled patients (EDSS > 3, *N* = 39), revealing that in the mild disability group, GCIPL thickness (*B* = 0.24, 95% CI = (0.03–0.45), *p* = 0.03), disease duration (*B* = −0.05, 95% CI = (0.27 to −0.02), *p* = 0.003) and years of education (*B* = 1.22, 95% CI = (0.27–2.17) *p* = 0.01) were significant predictors of SDMT scores. The model for this group explained significant variance in SDMT, *F*(5,84) = 5.64, *p* < 0.001, adjusted *R*^2^ = 0.21. In contrast, the analysis for the severely disabled group indicated no significant predictors (all *p* > 0.3) (see Table 5 in Supplemental Material (S3)).

Our second sensitivity analysis compared younger (<50) with older (>50) patients, showing that in the younger group, GCIPL thickness (*B* = 0.25, 95% CI = (9.91–0.50), *p* = 0.04), disease duration (*B* = −0.05, 95% CI = (−0.08 to −0.01), *p* = 0.006) and years of education (*B* = 1.38, 95% CI = (0.38–2.38), *p* = 0.007) were significant predictors of SDMT score. The overall model for younger patients explained significant variance in SDMT, *F*(5,93) = 4.37, *p* = 0.001, adjusted *R*^2^ = 0.15. Conversely, the analysis of the older group did not reveal any significant predictors (all *p* > 0.05), indicating a diminished influence of these factors on cognitive performance in this age group (see Table 5 in Supplemental Material (S3)).

## Discussion

In this cross-sectional study, we explored the association between OCT measures and BICAMS performance in 137 RRMS patients. In addition, we investigated the role of OCT as complementary to a standard clinical MRI protocol in explaining cognitive dysfunction in RRMS. We found that GCIPL was associated with SDMT performance and with WM and lesion volume. However, GCIPL did not contribute uniquely to predicting SDMT when MRI measures were also included. These findings suggest that OCT measures are relevant, but not unique predictors of cognitive dysfunction in RRMS.

Our study introduces several novel contributions that enhance the understanding of OCT in clinical monitoring for RRMS. First, we incorporated MRI metrics, which are well-established biomarkers of neuro-axonal damage and part of standard-of-care of MS, to assess the relative contributions of OCT and MRI measures. This provides a more comprehensive evaluation of these tools in cognitive dysfunction. Second, we used objective thresholds to define non-affected eyes, rather than relying on self-reported or clinically diagnosed optic neuropathy, which enhances the precision of our analysis. Third, our cohort exclusively comprises RRMS patients, unlike previous studies that included mixed clinical subtypes. These novel aspects strengthen the evidence for using OCT in monitoring clinical progression in RRMS, while showing that the established relationship between OCT metrics and clinical disability is not confounded by subclinical optic neuropathy.

Consistent with previous studies, we found the greatest impairment on the SDMT, a sensitive measure of cognitive efficiency in MS.^25^ We also found that 37.23% had visuospatial learning difficulties (BVMT-R) and only 5.11% had verbal learning deficits (CVLT-II).

We found a correlation between GCIPL and SDMT, which persisted when controlling for important confounders such as age, sex and disease duration. The initial correlation seen between RNFL and BVMT-R scores was lost after controlling for confounders. This is in line with previous findings that GCIPL showed stronger associations with cognition than pRNFL^7,11^ supporting the notion that GCIPL is advantageous over pRNFL in its reflection of atrophy.^26^ Our lack of associations with pRNFL contrasts with previous studies.^7,9,11^ Notably, most of these studies included older patients, longer disease durations or mixed clinical subtypes. Dreyer-Alster et al.^7^ found a significant association between RNFL and verbal function in a sample of 204 RRMS patients with similar age and disease duration to this study. We did not measure verbal function, and any associations with verbal memory and learning (CVLT-II) may have gone unnoticed due to the low impairment rate (5.11%). Among the studies that did not report associations, one used a younger cohort, with shorter disease duration and a lower physical disability,^12^ while another only investigated RNFL and not GCIPL.^13^ As our cohort positions itself clinically and demographically between those studies that found associations with both GCIPL and pRNFL, and those that did not, this likely implies that cognitive dysfunction precedes retinal thinning, or depend on different pathological aspects, as previously suspected.^13^

The results of our sensitivity analyses revealed that GCIPL thickness loses predictive power in patients with greater disability (EDSS > 3). This could indicate that in earlier MS, cognitive outcomes can be more effectively predicted by structural factors, while in more advanced stages, cognitive decline may be influenced by more complex, disease-related processes. The finding that GCIPL thickness was significant only in younger patients suggests that younger individuals may experience cognitive decline more directly related to MS-specific structural damage, while this association may be blurred by age-related cognitive decline in older patients. Nonetheless, it is important to acknowledge that the sample size of the severely disabled subcohort (*n* = 39) was much lower than the mildly disabled subcohort (*n* = 90), as was true for the young (*n* = 99) versus older (*n* = 30) subsamples. Therefore, we cannot exclude that these findings are owed to lack of statistical power in the smaller cohorts.

As expected, correlations between OCT measures and both cognitive and MRI measures were observed for eyes unaffected by ON, as ON-related thinning can confound OCT associations with degeneration.^27^

The association between GCIPL and SDMT did not persist when controlling for MRI measures (WM, GM and lesion volume). This contrasts the hypothesis that OCT may be a better correlate of neurodegeneration, as it is not confounded by myelin loss and ‘pseudoatrophy’, which are known limitations of conventional MRI. Nonetheless, this finding is unsurprising, as the association between OCT measures and clinical disability is thought to reflect brain atrophy.^5^ Our findings contrast with those reported by Cerdá-Fuertes et al.,^28^ where pRNFL remained a significant predictor of SDMT performance after controlling for brain parenchymal fraction. This inconsistency may be explained by differences in our samples: their cohort comprised both relapsing and progressive patients, with longer disease duration, older age and higher physical disability, and had a lower mean pRNFL value compared with the current cohort. It seems plausible that the complementary value of OCT next to MRI becomes apparent later in the disease, where the neurodegenerative component is more prominent.

While GM pathology is considered the prominent driver of cognitive dysfunction in MS, increasing evidence suggests that it might also be related to a network failure, starting with a decrease in WM integrity.^4^ In this respect, it is unsurprising that the reduced WM volume in the current cohort is the main predictor of performance on the SDMT, a sensitive although not specific measure of cognitive efficiency.^25^ SDMT performance is strongly associated with decrease in information processing speed; one of the main features of cognitive impairment in MS,^29^ associated with damage of both GM structures and their WM connections^30,31^ and their structural and functional changes.^32^ Moreover, the less prominent GM pathology observed in this study is not surprising given the middle-aged, relapsing-remitting cohort with a relatively short disease duration. It is possible that the potential unique variance explained by OCT relates more to GM pathology and is not evident in RRMS at an early disease stage. Thus, OCT may contribute uniquely to the detection of cognitive impairments in later, more progressive disease stages, where GM and WM damage becomes more prominent.

Our study has several strengths but also some limitations. First, as we aimed to investigate the potential role of OCT as a mirror of neurodegeneration, we employed strict criteria to ensure that the unaffected category was not contaminated with affected eyes by excluding eyes with a history of ON, a pRNFL above 75 µm and inter-eye differences of GCIPL => 4 µm and pRNFL => 5 µm, which are considered robust thresholds for identifying asymptomatic unilateral ON.^15^ Nonetheless, it is still possible that some patients had subclinical ON, which particularly in the case of bilateral subclinical ON, would not necessarily have been caught with the criteria applied. Furthermore, our approach to use OCT-based exclusion criteria (i.e., the IED and pRNFL absolute threshold) means that the results can be extrapolated only to patients with at least one unaffected optic nerve. In patients with a history of bilateral ON, the IED approach cannot be used to determine subclinical ON, and, therefore, it may only be possible to use the absolute pRNFL threshold to exclude patients with subclinical ON prior to using OCT metrics as biomarkers of CNS degeneration. Second, although the BICAMS represents a feasible measure currently recommended as a brief battery able to capture the most common cognitive deficits in MS,^16^ its use has limited the insight into other cognitive domains, such as executive functioning, that can be present since the time of diagnosis.^33^ Third, we did not control for depression or fatigue, which are prevalent in MS and may affect cognitive performance, although a clear link has not been demonstrated.^34^ Finally, a particular strength is that we controlled for visual acuity, which is known to influence performance on the SDMT and BVMT-R,^35^ thereby concluding that the reported associations between OCT and cognitive performance are not just reflective of visual impairments.

Overall, it appears that cognitive deficits can precede retinal thinning, possibly because of inflammation and network disruption, which can be better captured by MRI. This highlights the importance of monitoring cognition from the early stages of MS, where DMTs may alleviate inflammation and prevent neurodegeneration.

## Conclusion

While OCT appears to be a reliable predictor of cognitive efficiency as measured by the SDMT, even in early MS, it does not appear to explain variance beyond a standard clinical MRI protocol. It may be that both MRI and OCT represent similar markers of neurodegeneration. In clinical practice, where time and resources are limited, OCT may be most useful as an alternative monitoring strategy for those who are ineligible for MRI, or as a method of more frequent monitoring between MRIs, due to its ability to provide objective measurements quickly and inexpensively. GCIPL thickness may provide an accessible proxy of neurodegeneration and cognitive processing speed, without the need for post-processing. The advantages of OCT as complementary to MRI for longitudinal prediction and in determining MS subtypes with more prominent neurodegeneration remain to be elucidated.

## Supplemental Material

sj-docx-1-msj-10.1177_13524585241304356 – Supplemental material for Investigating the complementary value of OCT to MRI in cognitive impairment in relapsing-remitting multiple sclerosis Supplemental material, sj-docx-1-msj-10.1177_13524585241304356 for Investigating the complementary value of OCT to MRI in cognitive impairment in relapsing-remitting multiple sclerosis by Ronja Christensen, Amy Jolly, Charmaine Yam, Marios C Yiannakas, Ahmed T Toosy, Marco Pitteri, Anna He, Riccardo Nistri, Suraya Mohamud, Eirini Samdanidou, Alan J Thompson and Olga Ciccarelli in Multiple Sclerosis Journal
