# Supplementary material for: Investigating the complementary value of OCT to MRI in cognitive impairment in relapsing-remitting multiple sclerosis
Source: Mult Scler. 2024 Dec 20;31(2):218–30. doi: 10.1177/13524585241304356 (PMC11789427; doi:10.1177/13524585241304356)
Supplement: sj-docx-1-msj-10.1177_13524585241304356 – Supplemental material for Investigating the complementary value of OCT to MRI in cognitive impairment in relapsing-remitting multiple sclerosis [file sj-docx-1-msj-10.1177_13524585241304356.docx]

**Supplementary Material**

**S1: Magnetic Resonance Imaging Scanning Protocol**

MRI images were acquired on a 3 Tesla Phillips Ingenia CX 3T MRI system (Philips Medical Systems, Best, Netherlands) with the NV 16-channel coil. The brain MRI protocol comprised the following acquisitions for lesion volume and brain volume computation, respectively:

1. 3D T2-weighted Fluid Attenuation Inversion Recovery (3D-FLAIR) with repetition time (TR) = 4800 ms; echo time (TE) = 267 ms; inversion time (TI) = 1650 ms; field of view (FOV) = 256 x 256 mm^2^; voxel size = 1.2 x 1.2 x 1.2 mm^3^; number of excitations (NEX) = 2; SENSE factor phase/slice = 3/2; Turbo Spin Echo factor = 182; number of slices = 150; acquisition time = 3:36 min.
2. 3D T1-weighted magnetization prepared turbo field-echo (3D-TFE) sequence with TR = 7 ms; TE = 3.2 ms; TI = 823 ms; flip angle = 8°; FOV = 256 x256 mm^2^; voxel size = 1 x 1 x 1 mm^3^; NEX = 1; compressed SENSE factor = 6; Turbo Field Echo factor = 225; number of slices = 176; acquisition time = 1:56 min.

**S2: Optical Coherence Tomography Scanning Protocol**

Peripapillary RNFL scan (pRNFL): pRNFL was measured with a 3.45mm ring scan centred on the optic disc (40,000 A-scans; axial resolution 3.9µm; quality > 25; ART 100). The mean pRNFL of the four quadrants was used in the analysis.

Macular volume scan: Ganglion Cell Layer (GCL) and Inner Plexiform Layer (IPL) were measured using a macular volumetric scan centred on the fovea (20 X 20º volume; 73B scans; ART 25). The mean of the four quadrants of the GCL and the mean of the four quadrants of the IPL (3mm diameter cylinder of the 1-3-6mm ring) were added to form a total average Ganglion Cell-Inner Plexiform Layer (GCIPL) thickness measure.

The quality of scans was evaluated according to the OSCAR-IB Consensus Criteria for Retinal OCT Quality Assessment ^19^, automatically segmented, and manually corrected where necessary. Mean pRNFL and GCIPL values were subsequently used in the analysis.

**S3: Table 5 for Sensitivity Analysis**

**Table 5: Sensitivity Analyses for Mildly vs Severely Disabled and Younger vs Older Patients**

|  | Mildly disabled (N = 90) | Severely disabled (N = 39) | Young (N = 99) | Older (N = 30) |
| --- | --- | --- | --- | --- |
| GCIPL thickness | **B = 0.24**  **CI [0.03-0.45]**  **p = 0.03** | B = 0.23  CI [-0.26-0.72]  p = 0.35 | **B = 0.25**  **CI [0.01-0.50]**  **p = 0.04** | B = 0.11  CI [-0.35-0.58]  p = 0.62 |
| Age | B = -0.11  CI [-0.36-0.14]  p = 0.39 | B = -0.05  CI [-0.50-0.40]  p = 0.82 | B = -0.07  CI [-0.41-0.26]  p = 0.67 | B = 1.67  CI [-0.15-3.50]  p = 0.07 |
| Sex | B = 0.55  CI [-4.77-5.86]  p = 0.84 | B = -4.57  CI [-13-53-4.38]  p = 0.31 | B = -0.20  CI [-5.73-5.33]  p = 0.94 | B = -8.56  CI [-18.56-1.44]  p = 0.09 |
| Disease duration | **B = -0.05**  **CI [-0.08- -0.02]**  **p = 0.03** | B = 0.00  CI [-0.05-0.05]  p = 0.96 | **B = -0.05**  **CI [-0.08- -0.01]**  **p = 0.006** | B = -0.02  CI [-0.06-0.02]  p = 0.36 |
| Years of education | **B = 1.22**  **CI [0.27-2.17]**  **p = 0.01** | B = 0.74  CI [-0.87-2.36]  p = 0.36 | **B = 1.38**  **CI [0.38- 2.38]**  **p = 0.007** | B = 1.41  CI [-0.50-3.32]  p = 0.14 |
| Model estimates | **F(5,84) = 5.64, p < 0.001, adj. R^2^ = 0.21** | F(5,33) = 0.58, p = 0.72, adj. R^2^ = -0.06 | **F(5,93) = 4.37, p = 0.001, adj. R^2^ = 0.15** | F(5,24) = 1.47, p = 0.24, adj. R^2^ = 0.08 |

GCIPL: ganglion cell-inner plexiform layer.*Mild disability refers to an EDSS < 3, severe disability to EDSS > 3. Young patients refer to those aged under 50, while older patients are those over 50
